# 4-(4-Methyl­piperazin-1-yl)-3-(5-phenyl-1,3,4-oxadiazol-2-yl)-7-(trifluoro­meth­yl)quinoline

**DOI:** 10.1107/S1600536811044370

**Published:** 2011-10-29

**Authors:** Hoong-Kun Fun, Suhana Arshad, B. Garudachari, Arun M. Isloor, M. N. Satyanarayan

**Affiliations:** a X-ray Crystallography Unit, School of Physics, Universiti Sains Malaysia, 11800 USM, Penang, Malaysia; b Organic Electronics Division, Department of Chemistry, National Institute of Technology-Karnataka, Surathkal, Mangalore 575 025, India; c Department of Physics, National Institute of Technology-Karnataka, Surathkal, Mangalore 575 025, India

## Abstract

In the title compound, C_23_H_20_F_3_N_5_O, the piperazine ring adopts a chair conformation. The quinoline ring makes dihedral angles of 56.61 (11), 49.94 (12) and 42.58 (14)° with the piperazine ring, the 1,3,4-oxadiazole ring and the benzene ring, respectively. An intra­molecular C—H⋯O hydrogen bond generates an *S*(7) ring motif. In the crystal, mol­ecules are linked into infinite chains along the *b* axis by C—H⋯N hydrogen bonds.

## Related literature

For background to the properties and uses of quinoline deriv­atives, see: Kaur *et al.* (2010 ▶); Eswaran *et al.* (2010 ▶); Chou *et al.* (2010 ▶); Chen *et al.* (2004 ▶); Shingalapur *et al.* (2009 ▶). For ring conformations, see: Cremer & Pople (1975 ▶). For hydrogen-bond motifs, see: Bernstein *et al.* (1995 ▶). For bond-length data, see: Allen *et al.* (1987 ▶). 
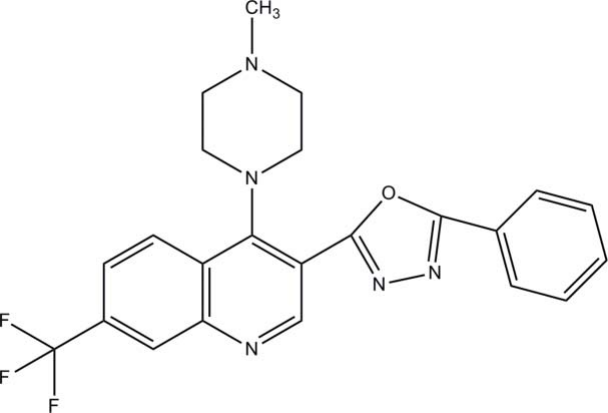


## Experimental

### 

#### Crystal data

C_23_H_20_F_3_N_5_O
*M* *_r_* = 439.44
Triclinic, 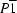

*a* = 8.5065 (15) Å
*b* = 10.2176 (17) Å
*c* = 13.709 (3) Å
α = 103.840 (5)°
β = 98.515 (5)°
γ = 109.034 (4)°
*V* = 1060.0 (4) Å^3^
*Z* = 2
Mo *K*α radiation
μ = 0.11 mm^−1^
*T* = 296 K
0.44 × 0.20 × 0.13 mm

#### Data collection

Bruker SMART APEXII DUO CCD diffractometer
Absorption correction: multi-scan (*SADABS*; Bruker, 2009 ▶) *T*_min_ = 0.954, *T*_max_ = 0.987
13724 measured reflections
4831 independent reflections
2890 reflections with *I* > 2σ(*I*)
*R*_int_ = 0.040

#### Refinement

*R*[*F* ^2^ > 2σ(*F* ^2^)] = 0.061
*wR*(*F* ^2^) = 0.231
*S* = 1.04
4831 reflections
290 parameters
H-atom parameters constrained
Δρ_max_ = 0.37 e Å^−3^
Δρ_min_ = −0.28 e Å^−3^

### 

Data collection: *APEX2* (Bruker, 2009 ▶); cell refinement: *SAINT* (Bruker, 2009 ▶); data reduction: *SAINT*; program(s) used to solve structure: *SHELXTL* (Sheldrick, 2008 ▶); program(s) used to refine structure: *SHELXTL*; molecular graphics: *SHELXTL*; software used to prepare material for publication: *SHELXTL* and *PLATON* (Spek, 2009 ▶).

## Supplementary Material

Crystal structure: contains datablock(s) global, I. DOI: 10.1107/S1600536811044370/hb6464sup1.cif

Structure factors: contains datablock(s) I. DOI: 10.1107/S1600536811044370/hb6464Isup2.hkl

Supplementary material file. DOI: 10.1107/S1600536811044370/hb6464Isup3.cml

Additional supplementary materials:  crystallographic information; 3D view; checkCIF report

## Figures and Tables

**Table 1:** Hydrogen-bond geometry (Å, °)

*D*—H⋯*A*	*D*—H	H⋯*A*	*D*⋯*A*	*D*—H⋯*A*
C21—H21*A*⋯O1	0.97	2.38	3.018 (3)	123
C4—H4*A*⋯N4^i^	0.93	2.56	3.426 (4)	155

Symmetry code: (i) 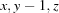.

**Fig. 1.**
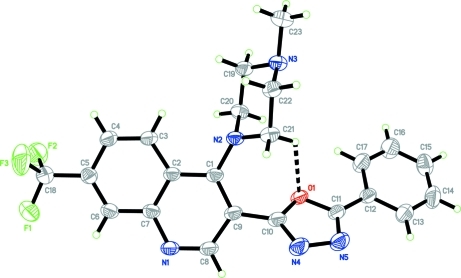
The molecular structure of the title compound, showing 30% probability displacement ellipsoids. The dashed line indicates the intramolecular bond.

**Fig. 2.**
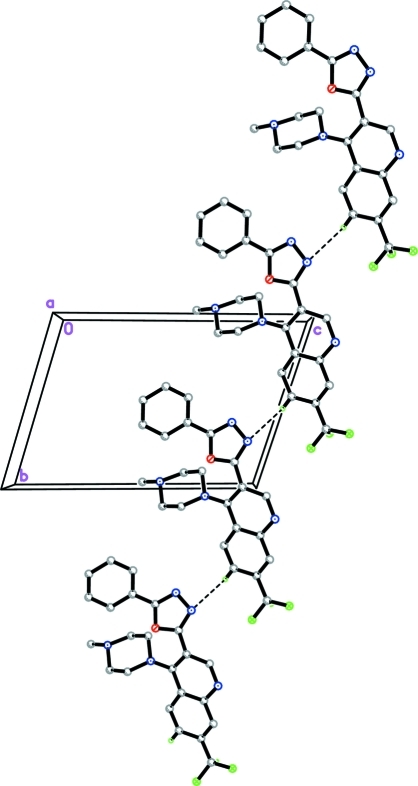
The crystal packing of the title compound. The dashed lines represent the hydrogen bonds. Hydrogen atoms not involved in hydrogen bonding have been omitted for the sake of clarity.

## supplementary crystallographic information

### Comment

The quinoline moiety is of great importance to chemists as well as biologists since it is one of the key building blocks for many naturally occurring compounds. Members of this family have wide range of applications in pharmaceuticals as antimalarial (Kaur *et al.*, 2010), anti-tuberculosis (Eswaran *et al.*, 2010), antitumor (Chou *et al.*, 2010), anticancer (Chen *et al.*, 2004) and antiviral (Shingalapur *et al.*, 2009) agents. Some of the present day drugs such as chloroquine, mefloquine, tafenoquine and primaquine carry the quinoline moiety as the basic unit in their structures. Keeping in view of these biological importance, we have synthesized the title compound to study its crystal structure.

The molecular structure is shown in Fig. 1. The piperazine ring adopts a chair conformation with puckering parameters Q= 0.586 (3) Å, Θ= 4.2 (3)° and φ= 259 (4)° (Cremer & Pople, 1975). The quinoline (N1/C1–C9) ring makes dihedral angles of 56.61 (11), 49.94 (12) and 42.58 (14)° with the piperazine ring (N2/N3/C19–C22), 1,3,4-oxadiazole ring (O1/N4/N5/C10/C11) and benzene ring (C12–C17), respectively. An intramolecular C21—H21A···O1 hydrogen bond (Table 1) stabilized the molecular structure and forms an *S*(7) ring motif (Bernstein *et al.*, 1995). Bond lengths (Allen *et al.*, 1987) and angles are within normal ranges.

In the crystal packing (Fig. 2), the molecules are linked into infinite one-dimensional chains along the *b*-axis by intermolecular C4—H4A···N4 hydrogen bonds (Table 1).

### Experimental

The mixture of 4-chloro-3-(5-phenyl-1,3,4-oxadiazol-2-yl)-7-(trifluoromethyl) quinoline (0.10 g, 0.00026 mol), potassium carbonate (0.040 g, 0.00029 mol) and 1-methylpiperazine (0.028 g, 0.00028 mol) in dimethylformamide (5 ml) was stirred at 90 °C for 5 h. After completion of the reaction, the reaction mixture was poured into ice-cold water. The solid product obtained was filtered, washed with water and recrystallized using ethanol to yield colouress blocks. Yield: 0.09 g; 77.58%. *M.p.*: 425–426 K.

### Refinement

All H atoms were positioned geometrically [C–H = 0.93, 0.96 or 0.97 Å] and refined using a riding model with *U*_iso_(H) = 1.2 or 1.5 *U*_eq_(C). A rotating group model was applied to the methyl group.

### Crystal data

**Table:**

C_23_H_20_F_3_N_5_O	*Z* = 2
*M**_r_* = 439.44	*F*(000) = 456
Triclinic, *P*1	*D*_x_ = 1.377 Mg m^−^^3^
Hall symbol: -P 1	Mo *K*α radiation, λ = 0.71073 Å
*a* = 8.5065 (15) Å	Cell parameters from 4219 reflections
*b* = 10.2176 (17) Å	θ = 2.3–26.9°
*c* = 13.709 (3) Å	µ = 0.11 mm^−^^1^
α = 103.840 (5)°	*T* = 296 K
β = 98.515 (5)°	Block, colourless
γ = 109.034 (4)°	0.44 × 0.20 × 0.13 mm
*V* = 1060.0 (4) Å^3^	

### Data collection

**Table:**

Bruker SMART APEXII DUO CCD diffractometer	4831 independent reflections
Radiation source: fine-focus sealed tube	2890 reflections with *I* > 2σ(*I*)
graphite	*R*_int_ = 0.040
φ and ω scans	θ_max_ = 27.5°, θ_min_ = 1.6°
Absorption correction: multi-scan (*SADABS*; Bruker, 2009)	*h* = −11→11
*T*_min_ = 0.954, *T*_max_ = 0.987	*k* = −13→12
13724 measured reflections	*l* = −17→17

### Refinement

**Table:**

Refinement on *F*^2^	Primary atom site location: structure-invariant direct methods
Least-squares matrix: full	Secondary atom site location: difference Fourier map
*R*[*F*^2^ > 2σ(*F*^2^)] = 0.061	Hydrogen site location: inferred from neighbouring sites
*wR*(*F*^2^) = 0.231	H-atom parameters constrained
*S* = 1.04	*w* = 1/[σ^2^(*F*_o_^2^) + (0.1328*P*)^2^ + 0.121*P*] where *P* = (*F*_o_^2^ + 2*F*_c_^2^)/3
4831 reflections	(Δ/σ)_max_ < 0.001
290 parameters	Δρ_max_ = 0.37 e Å^−^^3^
0 restraints	Δρ_min_ = −0.28 e Å^−^^3^

### Special details

**Table:**

Geometry. All e.s.d.'s (except the e.s.d. in the dihedral angle between two l.s. planes) are estimated using the full covariance matrix. The cell e.s.d.'s are taken into account individually in the estimation of e.s.d.'s in distances, angles and torsion angles; correlations between e.s.d.'s in cell parameters are only used when they are defined by crystal symmetry. An approximate (isotropic) treatment of cell e.s.d.'s is used for estimating e.s.d.'s involving l.s. planes.
Refinement. Refinement of *F*^2^ against ALL reflections. The weighted *R*-factor *wR* and goodness of fit *S* are based on *F*^2^, conventional *R*-factors *R* are based on *F*, with *F* set to zero for negative *F*^2^. The threshold expression of *F*^2^ > σ(*F*^2^) is used only for calculating *R*-factors(gt) *etc*. and is not relevant to the choice of reflections for refinement. *R*-factors based on *F*^2^ are statistically about twice as large as those based on *F*, and *R*- factors based on ALL data will be even larger.

### Fractional atomic coordinates and isotropic or equivalent isotropic displacement parameters (Å^2^)

**Table:**

	*x*	*y*	*z*	*U*_iso_*/*U*_eq_	
F1	0.5226 (3)	−0.6774 (2)	−0.27438 (12)	0.0963 (6)	
F2	0.5484 (3)	−0.7593 (2)	−0.14756 (14)	0.1015 (6)	
F3	0.7645 (3)	−0.6755 (2)	−0.20656 (17)	0.1229 (9)	
O1	0.7623 (2)	0.17853 (16)	0.22070 (11)	0.0530 (4)	
N1	0.6121 (3)	−0.1679 (2)	−0.10374 (15)	0.0627 (6)	
N2	0.9415 (2)	−0.0424 (2)	0.19566 (13)	0.0506 (5)	
N3	1.1662 (3)	0.0381 (2)	0.39233 (14)	0.0602 (5)	
N4	0.8354 (3)	0.2779 (3)	0.10041 (17)	0.0738 (7)	
N5	0.8316 (3)	0.3860 (2)	0.18536 (18)	0.0745 (7)	
C1	0.8270 (3)	−0.0818 (3)	0.09901 (16)	0.0487 (5)	
C2	0.7750 (3)	−0.2268 (3)	0.03114 (15)	0.0493 (5)	
C3	0.8327 (3)	−0.3329 (3)	0.05590 (17)	0.0580 (6)	
H3A	0.9059	−0.3092	0.1204	0.070*	
C4	0.7844 (3)	−0.4679 (3)	−0.01160 (18)	0.0606 (6)	
H4A	0.8240	−0.5357	0.0066	0.073*	
C5	0.6736 (3)	−0.5050 (3)	−0.10978 (17)	0.0555 (6)	
C6	0.6166 (3)	−0.4057 (3)	−0.13745 (17)	0.0585 (6)	
H6A	0.5446	−0.4314	−0.2027	0.070*	
C7	0.6652 (3)	−0.2655 (3)	−0.06883 (16)	0.0534 (6)	
C8	0.6647 (3)	−0.0358 (3)	−0.04098 (17)	0.0611 (6)	
H8A	0.6318	0.0313	−0.0652	0.073*	
C9	0.7691 (3)	0.0135 (3)	0.06230 (16)	0.0538 (6)	
C10	0.7947 (3)	0.1592 (3)	0.12490 (17)	0.0563 (6)	
C11	0.7883 (3)	0.3219 (3)	0.25302 (18)	0.0557 (6)	
C12	0.7691 (3)	0.3857 (3)	0.35531 (18)	0.0545 (6)	
C13	0.7751 (4)	0.5264 (3)	0.3839 (2)	0.0778 (8)	
H13A	0.7875	0.5794	0.3371	0.093*	
C14	0.7628 (5)	0.5883 (4)	0.4819 (3)	0.0946 (10)	
H14A	0.7675	0.6835	0.5011	0.113*	
C15	0.7436 (4)	0.5113 (3)	0.5516 (2)	0.0852 (9)	
H15A	0.7351	0.5539	0.6176	0.102*	
C16	0.7371 (5)	0.3730 (4)	0.5239 (2)	0.0907 (10)	
H16A	0.7241	0.3206	0.5710	0.109*	
C17	0.7497 (5)	0.3091 (3)	0.4258 (2)	0.0798 (8)	
H17A	0.7449	0.2139	0.4074	0.096*	
C18	0.6293 (4)	−0.6518 (3)	−0.1835 (2)	0.0661 (7)	
C19	1.0295 (3)	−0.1042 (3)	0.35015 (17)	0.0603 (6)	
H19A	0.9899	−0.1365	0.4063	0.072*	
H19B	1.0730	−0.1733	0.3137	0.072*	
C20	0.8802 (3)	−0.1007 (3)	0.27605 (16)	0.0568 (6)	
H20A	0.7941	−0.1983	0.2446	0.068*	
H20B	0.8279	−0.0404	0.3138	0.068*	
C21	1.0767 (3)	0.1013 (3)	0.23898 (18)	0.0585 (6)	
H21A	1.0344	0.1686	0.2788	0.070*	
H21B	1.1149	0.1372	0.1838	0.070*	
C22	1.2244 (3)	0.0892 (3)	0.30838 (18)	0.0599 (6)	
H22A	1.2661	0.0216	0.2682	0.072*	
H22B	1.3180	0.1834	0.3369	0.072*	
C23	1.3087 (4)	0.0320 (4)	0.4630 (2)	0.0857 (9)	
H23A	1.2690	0.0001	0.5184	0.129*	
H23B	1.3982	0.1269	0.4909	0.129*	
H23C	1.3524	−0.0351	0.4258	0.129*	

### Atomic displacement parameters (Å^2^)

**Table:**

	*U*^11^	*U*^22^	*U*^33^	*U*^12^	*U*^13^	*U*^23^
F1	0.1233 (15)	0.0904 (13)	0.0601 (9)	0.0372 (11)	0.0038 (9)	0.0121 (9)
F2	0.1358 (17)	0.0692 (11)	0.0916 (12)	0.0266 (10)	0.0267 (11)	0.0283 (9)
F3	0.0936 (14)	0.1201 (17)	0.1324 (17)	0.0514 (12)	0.0326 (12)	−0.0196 (13)
O1	0.0703 (10)	0.0585 (10)	0.0514 (8)	0.0397 (8)	0.0224 (7)	0.0284 (7)
N1	0.0840 (14)	0.0819 (15)	0.0464 (10)	0.0542 (12)	0.0177 (9)	0.0291 (10)
N2	0.0607 (11)	0.0572 (11)	0.0437 (9)	0.0274 (9)	0.0124 (8)	0.0261 (8)
N3	0.0700 (12)	0.0684 (13)	0.0479 (10)	0.0342 (11)	0.0073 (9)	0.0208 (9)
N4	0.1127 (19)	0.0686 (14)	0.0645 (13)	0.0471 (13)	0.0351 (12)	0.0373 (11)
N5	0.1079 (18)	0.0646 (14)	0.0703 (13)	0.0425 (13)	0.0323 (12)	0.0344 (12)
C1	0.0581 (12)	0.0623 (13)	0.0425 (10)	0.0336 (10)	0.0192 (9)	0.0266 (10)
C2	0.0592 (12)	0.0613 (14)	0.0431 (10)	0.0338 (11)	0.0173 (9)	0.0254 (10)
C3	0.0731 (15)	0.0656 (15)	0.0484 (11)	0.0387 (12)	0.0117 (10)	0.0252 (11)
C4	0.0770 (16)	0.0645 (15)	0.0567 (13)	0.0402 (13)	0.0194 (11)	0.0269 (12)
C5	0.0629 (13)	0.0649 (15)	0.0481 (11)	0.0301 (11)	0.0199 (10)	0.0217 (11)
C6	0.0650 (14)	0.0768 (17)	0.0448 (11)	0.0372 (12)	0.0144 (10)	0.0230 (11)
C7	0.0607 (13)	0.0724 (16)	0.0445 (11)	0.0381 (12)	0.0190 (9)	0.0267 (11)
C8	0.0831 (16)	0.0787 (18)	0.0496 (12)	0.0528 (14)	0.0231 (11)	0.0343 (12)
C9	0.0706 (14)	0.0669 (15)	0.0485 (11)	0.0436 (12)	0.0246 (10)	0.0305 (11)
C10	0.0721 (14)	0.0682 (15)	0.0508 (12)	0.0422 (12)	0.0233 (10)	0.0308 (11)
C11	0.0660 (13)	0.0553 (14)	0.0595 (13)	0.0337 (11)	0.0161 (10)	0.0263 (11)
C12	0.0576 (12)	0.0557 (13)	0.0597 (13)	0.0303 (11)	0.0139 (10)	0.0221 (11)
C13	0.104 (2)	0.0628 (17)	0.0746 (17)	0.0387 (15)	0.0225 (15)	0.0236 (14)
C14	0.133 (3)	0.0618 (18)	0.083 (2)	0.0410 (19)	0.0281 (19)	0.0046 (16)
C15	0.099 (2)	0.074 (2)	0.0714 (18)	0.0261 (16)	0.0282 (16)	0.0061 (15)
C16	0.138 (3)	0.080 (2)	0.0680 (17)	0.046 (2)	0.0438 (18)	0.0297 (15)
C17	0.123 (3)	0.0650 (18)	0.0700 (17)	0.0477 (17)	0.0387 (16)	0.0282 (14)
C18	0.0738 (16)	0.0691 (17)	0.0597 (14)	0.0324 (13)	0.0179 (12)	0.0188 (12)
C19	0.0815 (16)	0.0666 (16)	0.0456 (11)	0.0380 (13)	0.0145 (11)	0.0265 (11)
C20	0.0658 (14)	0.0656 (15)	0.0458 (11)	0.0251 (11)	0.0158 (10)	0.0273 (11)
C21	0.0611 (13)	0.0645 (15)	0.0606 (13)	0.0285 (11)	0.0154 (11)	0.0313 (12)
C22	0.0593 (13)	0.0658 (15)	0.0627 (14)	0.0310 (12)	0.0140 (11)	0.0242 (12)
C23	0.088 (2)	0.099 (2)	0.0671 (16)	0.0432 (17)	−0.0064 (14)	0.0253 (16)

### Geometric parameters (Å, °)

**Table:**

F1—C18	1.342 (3)	C8—C9	1.429 (3)
F2—C18	1.338 (3)	C8—H8A	0.9300
F3—C18	1.316 (3)	C9—C10	1.457 (3)
O1—C11	1.356 (3)	C11—C12	1.456 (3)
O1—C10	1.363 (3)	C12—C13	1.378 (4)
N1—C8	1.303 (3)	C12—C17	1.379 (4)
N1—C7	1.374 (3)	C13—C14	1.376 (4)
N2—C1	1.404 (3)	C13—H13A	0.9300
N2—C21	1.452 (3)	C14—C15	1.372 (4)
N2—C20	1.457 (3)	C14—H14A	0.9300
N3—C19	1.448 (3)	C15—C16	1.353 (5)
N3—C22	1.459 (3)	C15—H15A	0.9300
N3—C23	1.464 (3)	C16—C17	1.383 (4)
N4—C10	1.288 (3)	C16—H16A	0.9300
N4—N5	1.413 (3)	C17—H17A	0.9300
N5—C11	1.288 (3)	C19—C20	1.520 (3)
C1—C9	1.385 (3)	C19—H19A	0.9700
C1—C2	1.430 (3)	C19—H19B	0.9700
C2—C3	1.416 (3)	C20—H20A	0.9700
C2—C7	1.424 (3)	C20—H20B	0.9700
C3—C4	1.355 (3)	C21—C22	1.518 (3)
C3—H3A	0.9300	C21—H21A	0.9700
C4—C5	1.411 (3)	C21—H21B	0.9700
C4—H4A	0.9300	C22—H22A	0.9700
C5—C6	1.363 (3)	C22—H22B	0.9700
C5—C18	1.486 (4)	C23—H23A	0.9600
C6—C7	1.401 (4)	C23—H23B	0.9600
C6—H6A	0.9300	C23—H23C	0.9600
			
C11—O1—C10	103.18 (17)	C15—C14—C13	120.7 (3)
C8—N1—C7	117.4 (2)	C15—C14—H14A	119.6
C1—N2—C21	120.91 (17)	C13—C14—H14A	119.6
C1—N2—C20	119.14 (18)	C16—C15—C14	119.7 (3)
C21—N2—C20	111.94 (17)	C16—C15—H15A	120.2
C19—N3—C22	109.93 (18)	C14—C15—H15A	120.2
C19—N3—C23	110.1 (2)	C15—C16—C17	120.4 (3)
C22—N3—C23	110.4 (2)	C15—C16—H16A	119.8
C10—N4—N5	106.5 (2)	C17—C16—H16A	119.8
C11—N5—N4	105.9 (2)	C12—C17—C16	120.3 (3)
C9—C1—N2	124.0 (2)	C12—C17—H17A	119.8
C9—C1—C2	117.54 (19)	C16—C17—H17A	119.8
N2—C1—C2	118.30 (19)	F3—C18—F2	106.3 (3)
C3—C2—C7	117.7 (2)	F3—C18—F1	105.9 (2)
C3—C2—C1	123.64 (19)	F2—C18—F1	104.3 (2)
C7—C2—C1	118.6 (2)	F3—C18—C5	113.0 (2)
C4—C3—C2	122.0 (2)	F2—C18—C5	112.9 (2)
C4—C3—H3A	119.0	F1—C18—C5	113.7 (2)
C2—C3—H3A	119.0	N3—C19—C20	111.1 (2)
C3—C4—C5	119.5 (2)	N3—C19—H19A	109.4
C3—C4—H4A	120.3	C20—C19—H19A	109.4
C5—C4—H4A	120.3	N3—C19—H19B	109.4
C6—C5—C4	120.6 (2)	C20—C19—H19B	109.4
C6—C5—C18	121.3 (2)	H19A—C19—H19B	108.0
C4—C5—C18	118.0 (2)	N2—C20—C19	109.67 (19)
C5—C6—C7	120.8 (2)	N2—C20—H20A	109.7
C5—C6—H6A	119.6	C19—C20—H20A	109.7
C7—C6—H6A	119.6	N2—C20—H20B	109.7
N1—C7—C6	117.9 (2)	C19—C20—H20B	109.7
N1—C7—C2	122.6 (2)	H20A—C20—H20B	108.2
C6—C7—C2	119.4 (2)	N2—C21—C22	108.10 (19)
N1—C8—C9	124.8 (2)	N2—C21—H21A	110.1
N1—C8—H8A	117.6	C22—C21—H21A	110.1
C9—C8—H8A	117.6	N2—C21—H21B	110.1
C1—C9—C8	119.0 (2)	C22—C21—H21B	110.1
C1—C9—C10	124.4 (2)	H21A—C21—H21B	108.4
C8—C9—C10	116.4 (2)	N3—C22—C21	109.5 (2)
N4—C10—O1	111.9 (2)	N3—C22—H22A	109.8
N4—C10—C9	128.8 (2)	C21—C22—H22A	109.8
O1—C10—C9	119.1 (2)	N3—C22—H22B	109.8
N5—C11—O1	112.5 (2)	C21—C22—H22B	109.8
N5—C11—C12	127.8 (2)	H22A—C22—H22B	108.2
O1—C11—C12	119.6 (2)	N3—C23—H23A	109.5
C13—C12—C17	119.0 (2)	N3—C23—H23B	109.5
C13—C12—C11	119.9 (2)	H23A—C23—H23B	109.5
C17—C12—C11	121.0 (2)	N3—C23—H23C	109.5
C14—C13—C12	119.9 (3)	H23A—C23—H23C	109.5
C14—C13—H13A	120.1	H23B—C23—H23C	109.5
C12—C13—H13A	120.1		
			
C10—N4—N5—C11	−0.2 (3)	C8—C9—C10—N4	−47.0 (4)
C21—N2—C1—C9	−34.4 (3)	C1—C9—C10—O1	−46.5 (3)
C20—N2—C1—C9	112.0 (3)	C8—C9—C10—O1	128.1 (2)
C21—N2—C1—C2	141.4 (2)	N4—N5—C11—O1	−0.1 (3)
C20—N2—C1—C2	−72.2 (3)	N4—N5—C11—C12	178.8 (2)
C9—C1—C2—C3	176.0 (2)	C10—O1—C11—N5	0.3 (3)
N2—C1—C2—C3	−0.1 (3)	C10—O1—C11—C12	−178.7 (2)
C9—C1—C2—C7	−1.1 (3)	N5—C11—C12—C13	8.9 (4)
N2—C1—C2—C7	−177.17 (18)	O1—C11—C12—C13	−172.3 (2)
C7—C2—C3—C4	−1.0 (3)	N5—C11—C12—C17	−169.2 (3)
C1—C2—C3—C4	−178.1 (2)	O1—C11—C12—C17	9.6 (4)
C2—C3—C4—C5	0.1 (4)	C17—C12—C13—C14	0.4 (5)
C3—C4—C5—C6	0.7 (4)	C11—C12—C13—C14	−177.8 (3)
C3—C4—C5—C18	177.4 (2)	C12—C13—C14—C15	−0.4 (5)
C4—C5—C6—C7	−0.6 (4)	C13—C14—C15—C16	0.2 (6)
C18—C5—C6—C7	−177.2 (2)	C14—C15—C16—C17	0.0 (6)
C8—N1—C7—C6	−176.9 (2)	C13—C12—C17—C16	−0.3 (5)
C8—N1—C7—C2	−0.7 (3)	C11—C12—C17—C16	177.9 (3)
C5—C6—C7—N1	176.1 (2)	C15—C16—C17—C12	0.1 (6)
C5—C6—C7—C2	−0.2 (4)	C6—C5—C18—F3	117.0 (3)
C3—C2—C7—N1	−175.1 (2)	C4—C5—C18—F3	−59.7 (3)
C1—C2—C7—N1	2.2 (3)	C6—C5—C18—F2	−122.3 (3)
C3—C2—C7—C6	1.0 (3)	C4—C5—C18—F2	61.0 (3)
C1—C2—C7—C6	178.3 (2)	C6—C5—C18—F1	−3.8 (4)
C7—N1—C8—C9	−2.0 (4)	C4—C5—C18—F1	179.6 (2)
N2—C1—C9—C8	174.6 (2)	C22—N3—C19—C20	−57.9 (3)
C2—C1—C9—C8	−1.3 (3)	C23—N3—C19—C20	−179.7 (2)
N2—C1—C9—C10	−11.0 (4)	C1—N2—C20—C19	154.1 (2)
C2—C1—C9—C10	173.1 (2)	C21—N2—C20—C19	−56.7 (3)
N1—C8—C9—C1	3.0 (4)	N3—C19—C20—N2	55.0 (3)
N1—C8—C9—C10	−171.8 (2)	C1—N2—C21—C22	−151.8 (2)
N5—N4—C10—O1	0.4 (3)	C20—N2—C21—C22	59.7 (2)
N5—N4—C10—C9	175.7 (2)	C19—N3—C22—C21	60.9 (3)
C11—O1—C10—N4	−0.4 (3)	C23—N3—C22—C21	−177.4 (2)
C11—O1—C10—C9	−176.2 (2)	N2—C21—C22—N3	−61.0 (3)
C1—C9—C10—N4	138.5 (3)		

### Hydrogen-bond geometry (Å, °)

**Table:**

*D*—H···*A*	*D*—H	H···*A*	*D*···*A*	*D*—H···*A*
C21—H21A···O1	0.97	2.38	3.018 (3)	123
C4—H4A···N4^i^	0.93	2.56	3.426 (4)	155

Symmetry codes: (i) *x*, *y*−1, *z*.
